# A micromechanics based elasto-plastic damage model for unidirectional composites under off-axis tensile loads

**DOI:** 10.1038/s41598-020-57771-8

**Published:** 2020-01-21

**Authors:** Yanchao Wang, Dong Chen, Nengwen Li, Huanquan Yuan, Zengyu Zhu, Yongxiang Li, Zhengming Huang

**Affiliations:** 1 Automotive Engineering Institute, Guangzhou Automobile Group Co., Ltd, Guangzhou, 511434 China; 2 School of Aerospace Engineering & Applied Mechanics, Tongji University, Shanghai, 200092 China; 3 School of Mechanical and Automotive Engineering, South China University of Technology, Guangzhou, 510006 China; 4 Guangdong Yatai New Material Technology Co. Ltd, Guangzhou, 526241 China

**Keywords:** Composites, Mechanical properties

## Abstract

Nonlinear properties of composite materials are essential for their engineering application. In this work, a three-phase micromechanics bridging model is employed to evaluate the nonlinear behavior of a composite from properties of fiber, matrix and interphase. It is assumed that the matrix elastoplasticity and the interface damage are two major sources of the nonlinearity. The former is described by the J2 flow rule. The latter is approximated by an interphase with stiffness degradation. For an interphase, an equivalent damage stress is introduced to account for the effect of normal and shear stress on the interface damage growth. Further, an explicit empirical equation is developed to relate the equivalent damage stress and the stiffness degradation of an interphase. The present elasto-plastic damage model is validated by comparing with experimental data of a series of composites under off-axis tensile loads.

## Introduction

Fibrous composites have drawn great attention due to their excellent mechanical performance. A deep understanding of nonlinear behavior of composites is essential for their further application on lightweight design^1^. However, most engineering applications are limited to elastic range, since nonlinear analysis is relatively complex in theory and high cost in computation^2^. Thus, it is desirable to develop an explicit analytical and user-friendly model for the nonlinear analysis of composites.

Literatures^3,4^ show that the nonlinearity of a composite mainly results from the elastoplastic deformation of the matrix and the damage of fiber/matrix interface. For elasto-plastic deformation, many numerical and analytical models have been developed. Among those models, analytical micromechanics ones are more attractive owing to their high computational efficiency and lower experimental cost. For example, the incremental tangent model^5^, deformation secant model^6^, incremental-secant model^7^, isotropization Eshelby tensor approach^2^, etc. A comprehensive review concerning elastoplastic models can be found in Kanouté *et al*.^8^, Saeb *et al*.^9^ and Wang *et al*.^10,11^ However, it is reported that the predicted properties of such models are too stiff compared with experiment data^12^. Some researchers^13,14^ believe that it is because such models take the elasto-plastic deformation of a matrix as the only source of nonlinearity of a composite while the contribution of interface damage is ignored.

For the interface damage of composites, continuum damage models are often used. For example, Chang *et al*.^15^ developed a progressive damage model for a laminate with notched holes. Maimí *et al*.^16^ used a continuum damage model to evaluate the onset and evolution of inter-laminar failure of a composite laminate. O’Higgins *et al*.^17^ presented an experimental method to identify parameters required in a continuum damage model. However, their models are established in macro scale, meaning that a unidirectional composite layer is seen as homogeneous. Thus, the contribution of matrix and interface on the nonlinearity cannot be distinguished clearly. In addition, for such models, damage parameters have to be re-defined when the fiber volume fraction changed, which is of high cost in time and finance.

A practicable way is to combine the nonlinearity of matrix and interface damage in one model. Vogler *et al*.^18^ and Camanho *et al*.^19^ published a series of work regarding a transverse-isotropic elastoplastic model for fibrous polymer composites, where a non-associate plastic model and a smeared crack model are combined to describe the nonlinearity of a composite. However, models of Vogler *et al*.^18^, and Camanho *et al*.^19^ are established at meso-scale. Due to the nature of meso-scale models, no physical mechanism at micro scale can be revealed and the experimental cost is relative higher compared to micromechanics models. Some other researchers analyzed nonlinear behavior of composites at micro-scale, where a kind of plasticity, e.g. J2 flow rule, was applied for matrix elasto-plasticity and a cohesive element method is employed to simulate interface debonding. Examples can be found in the work of Melro *et al*.^20^, Han *et al*.^21^, and Pulungan *et al*.^22^ However, such models has to be implemented in a finite element model where no explicit expressions are provided. In addition, the computational cost of a cohesive element method is high as reported^13,23,24^. Hiremath *et al*.^25^ presented an analytical elasto-plastic damage micromechanics model for the stiffness reduction of a composite. Nevertheless, their work mainly focuses on stiffness degradation along fiber direction. It is still dubious whether the model is applicable for a composite under a complex load case. Zhao *et al*.^26^ proposed a plastic damage model for a composite at micro scale, where the damage of matrix is simulated by the variation of micro-crack density, and the plastic deformation is described by frictional sliding along micro-cracks. However, the interface damage is not accounted for in their model. Ju *et al*.^27^ developed a micromechanical elastoplastic damage model for fibrous composite. In their work, an orthotropic fiber is used to approximate a fiber with debonding interface. Ju *et al*.’s^27^ work didn’t consider the effect of shear stress on interface damage and is not applicable for a composite with an interphase.

In this work, an analytical micromechanics model is developed for the nonlinear analysis of unidirectional composites. Both the matrix elastoplasticity and interface damage are involved in this model. Firstly, the matrix elasto-plasticity is described by a J2 flow rule. Then, the interface damage is simulated by an interphase with stiffness degradation. A Mohr-Coulomb like criteria is presented to account for the effect of normal and shear stresses on the stiffness degradation of an interphase. Finally, an analytical three-phase bridging model^28^ is employed to predict mechanical properties of a composite from properties of fiber, matrix, and interphase. There are three advantages in this work. First, the model is computational efficiency owing to its explicit and analytical expressions. Second, analytical empirical equations are provided for the interphase degradation, where the normal- shear stress coupling effect on the interface damage is considered. Last, the present model is not only applicable for a composite with interface damage but also a composite with an interphase, such as fiber coatings.

This paper is organized as follows. Section 2 establishes a nonlinear model, in which the bridging model, interphase model, matrix elasto-plasticity, and failure criteria are introduced. An experimental validation is made in section 3. Conclusions are drawn in Section 4.

## Theory

**Figure 1:**
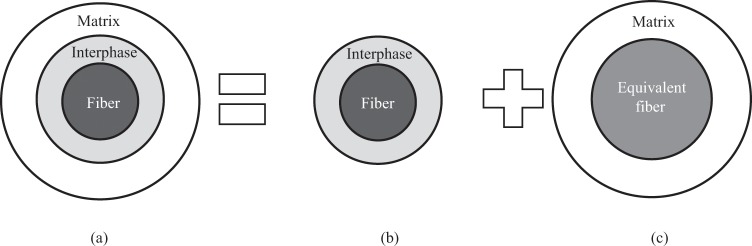
Equivalent fiber method for a three-phase composite. (**a**) Three-phase composite. (**b**) Inner two-phase composite. (**c**) Global two-phase composite.

Before introduce the bridging model in detail, proper nouns involved should be specified. The two-phase composite denotes a composite consisted of a fiber and a matrix. The three-phase composite refers to a composite containing an interphase in addition to a fiber and a matrix, as shown in Fig. 1(a). The inner two-phase composite represents a local structure made of a fiber and an interphase, as shown in Fig. 1(b). The global two-phase composite means a composite made of a matrix and an equivalent fiber, as shown in Fig. 1(c). The inner two-phase composite and the equivalent fiber are identical essentially but have different names for the convenience of presentation in different circumstances. Additionally, the noun “matrix” without an attributive adjective means the constituent material in a composite. In other cases, the noun “matrix” with a specified adjective, such as bridging matrix, stiffness matrix, means a mathematical rectangular array. Moreover, parameters with superscripts *f*, *c*, *ef*, and *m* denote quantities of a fiber, an interphase, an equivalent fiber, and a matrix. The notation description works throughout the paper.

### Two-phase bridging model

The original two-phase bridging model^29^ is established with a perfect interface assumption. Based on the bridging model, the effective compliance matrix of a unidirectional composite is given by Eq. (1).

1 $$[{M}_{ij}]=({V}^{f}[{M}_{ij}^{f}]+{V}^{m}[{M}_{ij}^{m}][{A}_{ij}]){({V}^{f}[{I}_{ij}]+{V}^{m}[{A}_{ij}])}^{-1},\,i,j=1,\,2,\,\ldots ,\,6$$

[*A*_*ij*_] is the bridging matrix. [*I*_*ij*_] is a six by six identity matrix. *V*^*f*^ and *V*^*m*^ are volume fractions of a fiber and a matrix in a composite. $[{M}_{ij}^{f}]$, $[{M}_{ij}^{m}]$, and [*M*_*ij*_] are, respectively, compliance matrix of a fiber, a matrix, and a composite.

Equation (2) shows the elements of [*M*_*ij*_] of a transversely isotropic composite in elastic range.

2 $$[{M}_{ij}]=[\begin{array}{cccccc}1/{E}_{11} & -{\upsilon }_{12}/{E}_{11} & -{\upsilon }_{12}/{E}_{11} & 0 & 0 & 0\\ -{\upsilon }_{12}/{E}_{11} & 1/{E}_{22} & -{\upsilon }_{23}/{E}_{22} & 0 & 0 & 0\\ -{\upsilon }_{12}/{E}_{11} & -{\upsilon }_{23}/{E}_{22} & 1/{E}_{22} & 0 & 0 & 0\\ 0 & \,0\, & 0 & 1/{G}_{23} & 0 & 0\\ 0 & 0 & 0 & 0 & 1/{G}_{12} & 0\\ 0 & 0 & 0 & 0 & 0 & 1/{G}_{12}\end{array}]$$

In a unidirectional fibrous composite laminate, direction “1” is parallel to a fiber. Direction “2” is in-plane and vertical to a fiber. Direction “3” is out-of-plane and through thickness. In eq. (2), *E*_11_ and *E*_22_ are longitudinal and transverse Young’s moduli of a composite, respectively. *G*_12_ and *G*_23_ are in-plane and out-of-plane shear moduli, respectively. ${\upsilon }_{12}$ and ${\upsilon }_{23}$ are in-plane and out-of-plane major Poison’s ratios. The compliance matrixes of a fiber and a matrix can be obtained by replacing parameters of a composite in Eq. (2) with their counterparts of a fiber and a matrix.

For a two-phase fibrous composite in elastic range, Huang^29^ presented detail information of the bridging matrix [*A*_*ij*_] as shown in Eqs. (3–7).

3 $$[{A}_{ij}]=[\begin{array}{cccccc}{A}_{11} & {A}_{12} & {A}_{13} & 0 & \,0\, & 0\\ 0 & {A}_{22} & 0 & 0 & 0 & 0\\ 0 & 0 & {A}_{33} & 0 & 0 & 0\\ 0 & \,0\, & 0 & {A}_{44} & 0 & 0\\ 0 & 0 & 0 & 0 & {A}_{55} & 0\\ 0 & 0 & 0 & 0 & 0 & {A}_{66}\end{array}]$$

4 $${A}_{11}=\frac{{E}_{\,}^{m}}{{E}_{11}^{f}}$$

5 $${A}_{22}={A}_{33}={A}_{44}=\beta +(1-\beta )\frac{{E}^{m}}{{E}_{22}^{f}},\beta =0.3 \sim 0.6$$

6 $${A}_{55}={A}_{66}=\alpha +(1-\alpha )\frac{{G}^{m}}{{G}_{12}^{f}},\alpha =0.3 \sim 0.6$$

7 $${A}_{12}={A}_{13}=({A}_{11}-{A}_{22})\frac{{M}_{12}^{f}-{M}_{12}^{m}}{{M}_{11}^{f}-{M}_{11}^{m}}$$

${E}_{11}^{f}$, ${E}_{22}^{f}$, ${G}_{12}^{f}$, ${M}_{11}^{f}$, ${M}_{12}^{f}$, and ${E}_{\,}^{m}$, ${G}_{\,}^{m}$, ${M}_{11}^{m}$, ${M}_{12}^{m}$ are, respectively Young’s moduli, shear moduli, and compliance matrix elements of a fiber and a matrix.

The concise bridging matrix, Eqs. (3–7), are obtained basing on a series expansion theory^29^. Please note that [*A*_*ij*_] is indeed non-symmetric and ${A}_{21}={A}_{31}={A}_{23}=0$. Wang *et al*.^30^ have proved that a rigorous bridging matrix can be derived from stress fields of a concentrated cylindrical assembly (CCA) model subjected to six different loads. The rigorous format of a bridging matrix is given in Eq. (8).

8 $$[{A}_{ij}^{r}]=[\begin{array}{cccccc}{A}_{11}^{r} & {A}_{12}^{r} & {A}_{13}^{r} & 0 & 0 & 0\\ {A}_{21}^{r} & {A}_{22}^{r} & {A}_{23}^{r} & 0 & 0 & 0\\ {A}_{31}^{r} & {A}_{32}^{r} & {A}_{33}^{r} & 0 & 0 & 0\\ 0 & 0 & 0 & {A}_{44}^{r} & 0 & 0\\ 0 & 0 & 0 & 0 & {A}_{55}^{r} & 0\\ 0 & 0 & 0 & 0 & 0 & {A}_{66}^{r}\end{array}]$$

The elements with superscript “*r*” denotes parameters derived rigorously. Wang *et al*.’s work^30^ shows that the bridging matrix is non-symmetric, and ${A}_{12}^{r}={A}_{13}^{r}$, ${A}_{21}^{r}={A}_{31}^{r}$, ${A}_{23}^{r}={A}_{32}^{r}$. In other words, the effective compliance matrix remains symmetric, although the bridging matrix is non-symmetric. Further, Wang *et al*.^30^ made a comparison between the rigorous bridging matrix and the Huang’s version. The conclusion is that the Huang’s version can be seen as a simplification of the rigorous one, when a fiber is much stiffer than a matrix in a composite, which is the common case of fiber reinforced epoxy composites.

Please note that Eqs. (3–7) are valid in elastic range. Based on the tangent linearization theory, mechanical properties of a material can be seen as linear in an infinitesimal load step. Thus, the bridging model can be extended to plastic range. However, the normal and shear stress may be coupled in plastic deformation. Strictly, the instantaneous bridging matrix in a load step should be calculated from an anisotropic Eshelby tensor^31^. However, such operation is too complex to be applied in engineering. Huang^29^ reported that the instantaneous bridging matrix can be rewritten as Eq. (9). The diagonal elements in Eq. (9) are given by substituting elastic parameters in Eqs. (3–6) with tangent instantaneous ones, as shown in Eqs. (10–12).

9 $$[{A}_{ij}^{t}]=[\begin{array}{cccccc}{A}_{11}^{t} & {A}_{12}^{t} & {A}_{13}^{t} & {A}_{14}^{t} & {A}_{15}^{t} & {A}_{16}^{t}\\ 0 & {A}_{22}^{t} & {A}_{23}^{t} & {A}_{24}^{t} & {A}_{25}^{t} & {A}_{26}^{t}\\ 0 & 0 & {A}_{33}^{t} & {A}_{34}^{t} & {A}_{35}^{t} & {A}_{36}^{t}\\ 0 & 0 & 0 & {A}_{44}^{t} & {A}_{45}^{t} & {A}_{46}^{t}\\ 0 & 0 & 0 & 0 & {A}_{55}^{t} & {A}_{56}^{t}\\ 0 & 0 & 0 & 0 & 0 & {A}_{66}^{t}\end{array}]$$

10 $${A}_{11}^{t}=\frac{{E}_{t}^{m}}{{E}_{11}^{f}}$$

11 $${A}_{22}^{t}={A}_{33}^{t}={A}_{44}^{t}=\beta +(1-\beta )\frac{{E}_{t}^{m}}{{E}_{22}^{f}},\beta =0.3 \sim 0.6$$

12 $${A}_{55}^{t}={A}_{66}^{t}=\alpha +(1-\alpha )\frac{{G}_{t}^{m}}{{G}_{12}^{f}},\alpha =0.3 \sim 0.6$$

Parameters with superscript or subscript *t* denote tangent instantaneous quantities. In each load step, the effective compliance matrix of a composite must be symmetric. The off-diagonal elements in Eq. (9) can be obtained by satisfying the symmetric condition shown in Eq. (13).

13 $$[{M}_{ij}^{t}]=[{M}_{ij}^{t}],\,i,{\rm{j}}=1,2,\ldots ,\,6$$

Formulation (13) provides fifteen equations, which is enough to solve all the fifteen off-diagonal elements in Eq. (9).

Equations (3) and (9) are the key of Huang’s elastoplastic micromechanics model, which has been validated by many researchers^32–34^. In this work, the Huang’s two-phase bridging model is employed to establish the three-phase bridging model.

### Three-phase bridging model

The two-phase bridging model is only valid for a composite with perfect interface. For a composite with interface damage, an imperfect interface condition must be considered. It is reported that an interphase model^35,36^ can approximate an imperfect interface. Thus, a three-phase CCA model as shown in Fig. 1(a) is employed for a composite with imperfect interface. Wang *et al*.^28^ gave an exact solution for a three-phase bridging matrix. Further, using an equivalent fiber method, a concise three-phase bridging model is available^37^.

Figure 1 illustrates the establishment process of a concise three-phase bridging model. Firstly, Take the fiber and interphase as an inner two-phase composite (Fig. 1(b)), where a two-phase bridging model is applicable. Regard the inner composite as an equivalent fiber. Then, a global two-phase composite (Fig. 1(c)) consisted of a matrix and an equivalent fiber is generated, where the two-phase bridging model can be applied once again.

Based on the bridging model, an effective compliance matrix of an inner two-phase composite is given by Eq. (14).

14 $$[{M}_{ij}^{ef}]=(\frac{{V}^{f}}{{V}^{f}+{V}^{c}}[{M}_{ij}^{f}]+\frac{{V}^{c}}{{V}^{f}+{V}^{c}}[{M}_{ij}^{c}][{A}_{ij}^{I}]){(\frac{{V}^{f}}{{V}^{f}+{V}^{c}}[{I}_{ij}]+\frac{{V}^{c}}{{V}^{f}+{V}^{c}}[{A}_{ij}^{I}])}^{-1},i,j=1,\,2,\,\ldots ,\,6$$

Again, the equivalent fiber and the inner two-phase composite are identical essentially. $[{M}_{ij}^{ef}]$ is the effective compliance matrix of an inner two-phase composite. $[{M}_{ij}^{f}]$ and $[{M}_{ij}^{c}]$ are, respectively, the compliance matrixes of a fiber and an interphase. [*I*_*ij*_] is a six by six identity matrix. *V*^*f*^ and *V*^*c*^ are corresponding volume fractions of a fiber and an interphase in a three-phase composite model. $[{A}_{ij}^{I}]$ is an instantaneous two-phase bridging matrix for an inner composite. The elements of $[{A}_{ij}^{I}]$ are given by^37^ Eqs. (15–19).

15 $$[{A}_{ij}^{I}]=[\begin{array}{cccccc}{A}_{11}^{I} & {A}_{12}^{I} & {A}_{13}^{I} & 0 & 0 & 0\\ 0 & {A}_{22}^{I} & 0 & 0 & 0 & 0\\ 0 & 0 & {A}_{33}^{I} & 0 & 0 & 0\\ 0 & 0 & 0 & {A}_{44}^{I} & 0 & 0\\ 0 & 0 & 0 & 0 & {A}_{55}^{I} & 0\\ 0 & 0 & 0 & 0 & 0 & {A}_{66}^{I}\end{array}]$$

16 $${A}_{11}^{I}=\frac{{E}_{\,}^{c}}{{E}_{11}^{f}}$$

17 $${A}_{22}^{I}={A}_{33}^{I}={A}_{44}^{I}=\beta +(1-\beta )\frac{{E}_{\,}^{c}}{{E}_{22}^{f}},\,\beta =0.3 \sim 0.6$$

18 $${A}_{55}^{I}={A}_{66}^{I}=\alpha +(1-\alpha )\frac{{G}_{\,}^{c}}{{G}_{12}^{f}},\,\alpha =0.3 \sim 0.6$$

19 $${A}_{12}^{I}={A}_{13}^{I}=({A}_{11}^{I}-{A}_{22}^{I})\frac{{M}_{12}^{f}-{M}_{12}^{c}}{{M}_{11}^{f}-{M}_{11}^{c}}$$

${E}_{\,}^{c}$ and ${G}_{\,}^{c}$ are Young’s and shear moduli of an interphase. ${E}_{11}^{f}$, ${E}_{22}^{f}$, ${G}_{12}^{f}$ are longitudinal Young’s, transverse Young’s, and longitudinal shear moduli of a fiber, respectively. ${M}_{11}^{c}$, ${M}_{12}^{c}$, and ${M}_{11}^{f}$, ${M}_{12}^{f}$ are elements in compliance matrixes of an interphase and a fiber, respectively*. α* and *β* in Eqs. (17) and (18) are bridging parameters which can be adjusted by experiments.

For a global two-phase composite, the effective compliance matrix is given as Eq. (20)

20 $$[{M}_{ij}^{\,}]=(({V}^{f}+{V}^{c})[{M}_{ij}^{ef}]+{V}^{m}[{M}_{ij}^{m}]\,[{A}_{ij}^{II}]){(({V}^{f}+{V}^{c})[{I}_{ij}]+{V}^{m}[{A}_{ij}^{II}])}^{-1}$$

where [*M*_*ij*_] is the effective compliance matrix of a global two-phase composite. $[{M}_{ij}^{m}]$ and $[{M}_{ij}^{ef}]$ are the compliance matrixes of a matrix and an equivalent fiber, respectively. $[{A}_{ij}^{II}]$ is a bridging matrix for a global two-phase composite, whose expressions are given in Eqs. (21–25).

21 $$[{A}_{ij}^{II}]=[\begin{array}{cccccc}{A}_{11}^{II} & {A}_{12}^{II} & {A}_{13}^{II} & 0 & 0 & 0\\ 0 & {A}_{22}^{II} & 0 & 0 & 0 & 0\\ 0 & 0 & {A}_{33}^{II} & 0 & 0 & 0\\ 0 & 0 & 0 & {A}_{44}^{II} & 0 & 0\\ 0 & 0 & 0 & 0 & {A}_{55}^{II} & 0\\ 0 & 0 & 0 & 0 & 0 & {A}_{66}^{II}\end{array}]$$

22 $${A}_{11}^{II}=\frac{{E}^{m}}{{E}_{11}^{ef}}$$

23 $${A}_{22}^{II}={A}_{33}^{II}={A}_{44}^{II}=\beta +(1-\beta )\frac{{E}^{m}}{{E}_{22}^{ef}},\,\beta =0.3\, \sim \,0.6$$

24 $${A}_{55}^{II}={A}_{66}^{II}=\alpha +(1-\alpha )\frac{{G}^{m}}{{G}_{12}^{ef}},\,\alpha =0.3\, \sim \,0.6$$

25 $${A}_{12}^{II}={A}_{13}^{II}=({A}_{11}^{II}-{A}_{22}^{II})\frac{{M}_{12}^{ef}-{M}_{12}^{m}}{{M}_{11}^{ef}-{M}_{11}^{m}}$$

*E*^*m*^ and *G*^*m*^ are Young’s and shear moduli of a matrix. ${E}_{11}^{ef}$, ${E}_{22}^{ef}$ and ${G}_{12}^{ef}$ are longitudinal Young’s, transverse Young’s, and longitudinal shear moduli of an equivalent fiber, whose expressions are given by Eq. (26).

26 $${E}_{11}^{ef}=\frac{1}{{M}_{11}^{ef}},\,{E}_{22}^{ef}=\frac{1}{{M}_{22}^{ef}},\,{G}_{12}^{ef}=\frac{1}{{M}_{66}^{ef}}$$

${M}_{11}^{m}$, ${M}_{12}^{m}$, and ${M}_{11}^{ef}$, ${M}_{12}^{ef}$, ${M}_{66}^{ef}$ are elements in the compliance matrixes of a matrix and an equivalent fiber.

Then, the stresses of each phase in a three-phase composite can be calculated from subjected external loads. For nonlinear analysis, formulations are in incremental forms as shown in Eqs. (27–30).

27 $$\{d{\sigma }_{i}^{m}\}=[{A}_{ij}^{II}]{(({V}^{f}+{V}^{c})[{I}_{ij}]+{V}^{m}[{A}_{ij}^{II}])}^{-1}\{d{\sigma }_{j}^{\,}\}$$

28 $$\{d{\sigma }_{i}^{ef}\}={(({V}^{f}+{V}^{c})[I]+{V}^{m}[{A}_{ij}^{II}])}^{-1}\{d{\sigma }_{j}\}$$

29 $$\{d{\sigma }_{i}^{c}\}=[{A}_{ij}^{I}]{({V}_{f}^{ef}[{I}_{ij}]+{V}_{c}^{ef}[{A}_{ij}^{I}])}^{-1}\{d{\sigma }_{j}^{ef}\}$$

30 $$\{d{\sigma }_{i}^{f}\}={({V}_{f}^{ef}[{I}_{ij}]+{V}_{c}^{ef}[{A}_{ij}^{I}])}^{-1}\{d{\sigma }_{j}^{ef}\}$$

where $$\{d{\sigma }_{j}\}=\{\begin{array}{cc}\begin{array}{ccc}d{\sigma }_{11} & d{\sigma }_{22} & d{\sigma }_{33}\end{array} & \begin{array}{ccc}d{\sigma }_{23} & d{\sigma }_{13} & d{\sigma }_{12}\end{array}\end{array}\}$$ is a contract incremental stress tensor of the external load subjected to a composite. Similarly, $\{d{\sigma }_{i}^{f}\}$, $\{d{\sigma }_{i}^{c}\}$, $\{d{\sigma }_{i}^{m}\}$, and $\{d{\sigma }_{i}^{ef}\}$ are corresponding stress increments of a fiber, an interphase, a matrix, and an equivalent fiber in a composite. Since constitutive equations are built in incremental forms for each infinitesimal load step, all the mechanical properties of constituent materials involved in Eqs. (14–30) are instantaneous.

### Elasto-plasticity of matrix

The elastoplastic deformation of a matrix is one main source of the total nonlinearity of a composite. J2 flow rule is a well-known plastic flow rule that applicable for both metal and nonmetal materials. When an isotropic work hardening law and the J2 flow rule is used, the instantaneous compliance matrix of a matrix material is given by Eq. (31).

31 $$[{M}_{ij}^{m}]={[{M}_{ij}^{m}]}^{e}+{[{M}_{ij}^{m}]}^{p}\,$$

where ${[{M}_{ij}^{m}]}^{e}$ and ${[{M}_{ij}^{m}]}^{p}$ are, respectively, elastic and plastic parts of the compliance matrix, whose expressions are shown in Eqs. (32–34).

32 $${[{M}_{ij}^{m}]}^{e}=[\begin{array}{cc}\begin{array}{ccc}1/{E}^{m} & -{\upsilon }^{m}/{E}^{m} & -{\upsilon }^{m}/{E}^{m}\\ \, & 1/{E}^{m} & -{\upsilon }^{m}/{E}^{m}\\ \, & \, & 1/{E}^{m}\end{array} & \begin{array}{ccc}0 & \,0\, & 0\\ 0 & 0 & 0\\ 0 & 0 & 0\end{array}\\ \begin{array}{ccc}\, & symmetric & \,\end{array} & \begin{array}{ccc}1/{G}^{m} & 0 & 0\\ \, & 1/{G}^{m} & 0\\ \, & \, & 1/{G}^{m}\end{array}\end{array}]$$

33 $${[{M}_{ij}^{m}]}^{p}={N}_{T}[\begin{array}{cccccc}{\sigma }_{11}^{m^{\prime} }{\sigma }_{11}^{m^{\prime} } & {\sigma }_{22}^{m^{\prime} }{\sigma }_{11}^{m^{\prime} } & {\sigma }_{33}^{m^{\prime} }{\sigma }_{11}^{m^{\prime} } & {\sigma }_{23}^{m^{\prime} }{\sigma }_{11}^{m^{\prime} } & {\sigma }_{13}^{m^{\prime} }{\sigma }_{11}^{m^{\prime} } & {\sigma }_{12}^{m^{\prime} }{\sigma }_{11}^{m^{\prime} }\\  & {\sigma }_{22}^{m^{\prime} }{\sigma }_{22}^{m^{\prime} } & {\sigma }_{33}^{m^{\prime} }{\sigma }_{22}^{m^{\prime} } & {\sigma }_{23}^{m^{\prime} }{\sigma }_{22}^{m^{\prime} } & {\sigma }_{13}^{m^{\prime} }{\sigma }_{22}^{m^{\prime} } & {\sigma }_{12}^{m^{\prime} }{\sigma }_{22}^{m^{\prime} }\\  &  & {\sigma }_{33}^{m^{\prime} }{\sigma }_{33}^{m^{\prime} } & {\sigma }_{23}^{m^{\prime} }{\sigma }_{33}^{m^{\prime} } & {\sigma }_{13}^{m^{\prime} }{\sigma }_{33}^{m^{\prime} } & {\sigma }_{12}^{m^{\prime} }{\sigma }_{33}^{m^{\prime} }\\  &  &  & {\sigma }_{23}^{m^{\prime} }{\sigma }_{23}^{m^{\prime} } & {\sigma }_{13}^{m^{\prime} }{\sigma }_{23}^{m^{\prime} } & {\sigma }_{12}^{m^{\prime} }{\sigma }_{23}^{m^{\prime} }\\  & symmetric &  &  & {\sigma }_{13}^{m^{\prime} }{\sigma }_{13}^{m^{\prime} } & {\sigma }_{12}^{m^{\prime} }{\sigma }_{13}^{m^{\prime} }\\  &  &  &  &  & {\sigma }_{12}^{m^{\prime} }{\sigma }_{12}^{m^{\prime} }\end{array}]$$

34 $${N}_{T}=\frac{9({E}^{m}-{E}_{t}^{m})}{4{({\sigma }_{eq}^{m})}^{2}{E}^{m}{E}_{t}^{m}}$$

The deviatoric stresses ${\sigma }_{ij}^{m^{\prime} },i,j=1,2,3$ in Eq. (33) are given in Eq. (35).

35 $${\sigma }_{ij}^{m^{\prime} }={\sigma }_{ij}^{m}-\frac{{\sigma }_{11}^{m}+{\sigma }_{22}^{m}+{\sigma }_{33}^{m}}{3}{\delta }_{ij},\,\,{\delta }_{ij}=\{\begin{array}{cc}0, & \,if\,i\ne j\\ 1, & \,if\,i=j\end{array},\,{\rm{i}},{\rm{j}}=1,2,3$$

${E}_{t}^{m}$ in Eq. (34) is a tangential Young’s moduli of a matrix. When an isotropic work hardening law is used, ${E}_{t}^{m}$ is given by Eq. (36).

36 $${E}_{t}^{m}=\{\begin{array}{ll}{E}^{m},\, & if\,{\sigma }_{eq}^{m}\le {\sigma }_{Y}^{m}\,\\ {E}_{t}^{m,n}, & if\,{\sigma }_{Y}^{m,\,n-1} < {\sigma }_{eq}^{m}\le {\sigma }_{Y}^{m,n},n\ge 1\end{array}$$

${E}_{T}^{m,n}$ is the tangential Young’s modulus at the *n*^th^ load step. ${\sigma }_{Y}^{m}$ is the initial tensile yielding strength of a matrix. ${\sigma }_{Y}^{m,n}$ is the tensile yielding strength at the *n*^th^ load step. ${\sigma }_{eq}^{m}$ is the von-Mises stress of a matrix, whose expression is shown in Eq. (37).

37 $${\sigma }_{eq}^{m}=\frac{1}{\sqrt{2}}\sqrt{{({\sigma }_{11}^{m}-{\sigma }_{22}^{m})}^{2}+{({\sigma }_{22}^{m}-{\sigma }_{33}^{m})}^{2}+{({\sigma }_{11}^{m}-{\sigma }_{33}^{m})}^{2}+6({({\sigma }_{12}^{m})}^{2}+{({\sigma }_{13}^{m})}^{2}+{({\sigma }_{23}^{m})}^{2})}$$

${E}_{t}^{m,n}$ can be obtained by a uniaxial test of a matrix. Alternatively, it can also be obtained from a tangential shear modulus, as shown in Eq. (38).

38 $${E}_{t}^{m}=2{G}_{t}^{m}(1+{\upsilon }_{t}^{m})\,$$

In plastic deformation, the Poisson’s ratio ${\upsilon }_{t}^{m}$ can be set as 0.5, according to the non-compressive law. The tangential shear modulus, ${G}_{t}^{m}$, is given by Eq. (39).

39 $${G}_{t}^{m}=\{\begin{array}{ll}{G}^{m}, & if\,{\tau }_{eq}^{m}\le {\tau }_{Y}^{m}\,\\ {G}_{t}^{m,n}, & \,if\,{\tau }_{Y}^{m,\,n-1} < {\tau }_{eq}^{m}\le {\tau }_{Y}^{m,n},\,n\ge 1\end{array}$$

${\tau }_{Y}^{m}$ is the initial shear yielding strength. ${\tau }_{Y}^{m,n}$ and ${G}_{T}^{m,n}$ are, respectively, the shear yielding strength and shear tangential modulus at the *n*^th^ load step. ${\tau }_{eq}^{m}$ is the octahedral shear stress of a matrix, whose expression is given in Eq. (40).

40 $${\tau }_{eq}^{m}=\frac{1}{\sqrt{6}}\sqrt{{({\sigma }_{11}^{m}-{\sigma }_{22}^{m})}^{2}+{({\sigma }_{22}^{m}-{\sigma }_{33}^{m})}^{2}+{({\sigma }_{11}^{m}-{\sigma }_{33}^{m})}^{2}+6({({\sigma }_{12}^{m})}^{2}+{({\sigma }_{13}^{m})}^{2}+{({\sigma }_{23}^{m})}^{2})}$$

Since the shear nonlinear behavior is more significant than tension, it is better to use a shear stress-strain curve when available.

### Interphase model

Nonlinear behavior induced by interface damage is more significant than longitudinal or transverse loads for a composite under off-axis tension. Thus, this work focuses on a UD composite subjected to an off-axis tension load. The case of off-axis compression would be investigated in future work.

Many researchers^35,36^ pointed out that the interface damage can be approximated by an interphase with stiffness degradation. Following their suggestion, a three-phase CCA model is utilized to simulate a composite with interface damage in this work. In general, degraded stiffness of an interphase must be determined experimentally. However, more parameters lead to higher experimental cost. One goal of our model is to reduce the parameter amount in an interphase model. Theoretically, in each infinitesimal load step, an off-axis load subjected to a UD composite can be seen as a combination of longitudinal normal stress, transverse normal stress, and longitudinal shear stress. It is well known that a UD composite is nearly linear elastic under a longitudinal tensile/compressive or transverse tensile load. For transverse compressive load, a UD composite is nonlinear to some extent. However, it majorly results from the matrix plasticity instead of interface damage. Some reports^4,38,39^ show that interface damage plays a key role in shear nonlinear deformation. Thus, for off-axis tensile load considered in this work, only the shear modulus of an interphase *G*^*c*^ is assumed to depend on the damage states while the Young’s modulus *E*^*c*^ remains constant.

Following the continuum damage theory, the compliance matrix of an interphase is given as Eq. (38).

41 $${M}_{D}^{c}=[\begin{array}{llllll}1/{E}^{c} & -{\upsilon }^{c}/{E}^{c} & -{\upsilon }^{c}/{E}^{c} & 0 & 0 & 0\\  & 1/{E}^{c} & -{\upsilon }^{c}/{E}^{c} & 0 & 0 & 0\\  &  & 1/{E}^{c} & 0 & 0 & 0\\  &  &  & 1/{G}_{D}^{c} & 0 & 0\\  &  &  &  & 1/{G}_{D}^{c} & 0\\  & symmetric &  &  &  & 1/{G}_{D}^{c}\end{array}]$$

where *E*^*c*^ and *υ*^*c*^ are, respectively, elastic Young’s modulus and Poisson ratio of an interphase. ${G}_{D}^{c}$ is a shear damage modulus whose values are sensitive to the interphase damage state.

**Figure 2:**
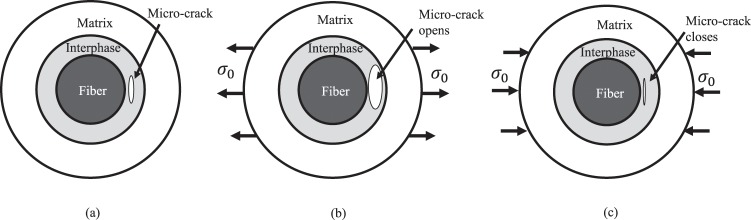
Effect of loads on micro-crack propagation. (**a**) An interphase with a micro-crack. (**b**) Micro-crack opens due to tension. (**c**) Micro-crack contracts due to compression.

Ideally, the shear damage modulus ${G}_{D}^{c}$ is majorly controlled by shear stress in an interphase. However, experimental results^40^ show that transverse normal stress can also affect the evolution of ${G}_{D}^{c}$. Approximately, a damaged interface can be simulated by an interphase with micro-cracks, as shown in Fig. 2. The micro-cracks expand or contract when a composite subjected to transverse tension or compression. In other words, transverse tension accelerates while transverse compression retards the micro-crack propagation, thereby affecting the stiffness degradation of an interphase. To consider the interaction between normal and shear stress, the Mohr-Coulomb failure theory is proposed for soil material^41^. Further, it is widely used in analysis of matrix plasticity^42^, interface damage^43^, and composite failure^40^. In this work, a Mohr-Coulomb-like damage criterion is developed as shown in Eqs. (42) and (43) to determine ${G}_{D}^{c}$, with which the normal-shear stress coupling effect on the damage evolution of an interphase can be considered.

42 $$\,{f}_{G}^{c}=\sqrt{{({\tau }_{12}^{c}+b{\sigma }_{22}^{c})}^{2}}$$

43 $${G}_{D}^{c}=\{\begin{array}{cc}{G}_{D}^{c,n}, & if\,{f}_{G}^{c}\le {f}_{G}^{c,n}\\ {G}_{D}^{c,n+1}, & if\,{f}_{G}^{c} > {f}_{G}^{c,n}\end{array}$$

where ${G}_{D}^{c,n}$ and ${G}_{D}^{c,n+1}$ are shear damage moduli for the *n*^*th*^ and (*n* + 1)^*th*^ load step, respectively. ${f}_{G}^{c}$ is the calculated driving stress of the shear damage modulus. ${f}_{G}^{c,n}$ is the experimental determined driving stress in the *n*^*th*^ load step. *b* is a positive coupling parameter between shear and normal stress, which is determined by experiments. From Eq. (42), it is found that a positive ${\sigma }_{22}^{c}$ will increase the value of ${f}_{G}^{c}$, thereby accelerating the damage process. When ${\sigma }_{22}^{c}$ is negative, the ${f}_{G}^{c}$ will be reduced, meaning that the damage process is delayed. Thus, Eq. (42) is able to consider the effect of a transverse normal stress on the shear damage process. Equation (43) is the evolution law for the shear modulus of an interphase, where the damage driving stress is employed as a criterion.

**Figure 3:**
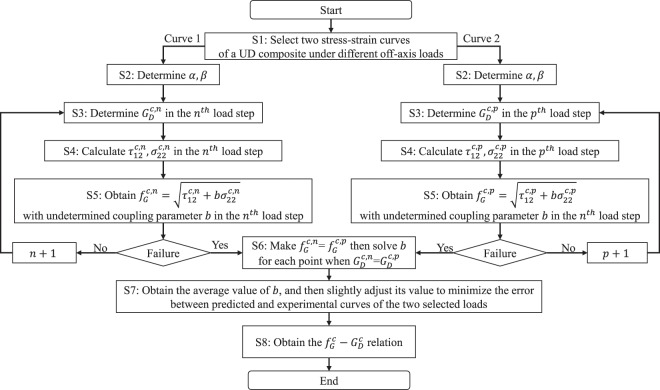
Engineering reverse process for the interphase properties.

It is very difficult to measure the mechanical properties of an interphase in a composite. Alternatively, engineering reverse calculation is a proper way. Figure 3 illustrates the reverse calculation process. Two parameters, ${G}_{D}^{c}$ and *b*, need to be reverse calculated in each load step, thereby requiring two experimental stress-strain curves. Thus, step one it to select two experimental stress-strain curves of a UD composite under different off-axis loads. Step two, determine bridging parameters, α and β. Strictly, the two parameters should be determined by fitting experimental data. If no experimental result is available, ${\rm{\alpha }}={\rm{\beta }}=0.3$ is recommended. According to Huang’s work^29^, the two bridging parameters keeps unchanged throughout the loading process. Step three, calculate the effective property of a composite by the three-phase bridging model from properties of constituent materials. Then, adjust ${G}_{D}^{c,n}$ to make the predicted effective properties agree with experimental data in each load step. Step five, calculate the shear stress and transverse normal stress in each load step. Then, get the expression of ${f}_{G}^{c,n}$ with an undetermined coupling parameter *b*. Continue step three to step five until failure. Step six, list ${G}_{D}^{c}$ curve fitted from two experimental stress-strain curves. Theoretically, for a monotonous load case, the damage driving stress and the damaged shear modulus should be one-to one mapping. Thus, the coupling parameter *b* can be solved by making ${f}_{G}^{c,n}={f}_{G}^{c,p}$ for each point when ${G}_{D}^{c,n}={G}_{D}^{c,p}$. The superscripts *n* and *p* represent the ${n}^{th}$ and ${p}^{th}$ load step of two selected off-axis loads. Step seven, obtain the average value of *b*, and slightly adjust its value to minimize the error between the predicted and experimental results. Finally, a relation between ${f}_{G}^{c}$ and ${G}_{D}^{c}$ is built to describe the mechanical behavior of an interphase.

Please note that, according to Huang’s work^29^, α and β keep unchanged throughout a load case. When experimental data is available, the value of α and β should be adjusted by fitting the elastic properties of a composite instead of setting α = β = 0.3. If α and β are not well-determined, the error resulting from elastic range will be transferred to nonlinear part, making the curve fitted interphase properties may deviate from reasonable values to some extent. Besides, the model shown in Eqs. (42, 43) is built based on a basic assumption that the shear damage modulus ${G}_{D}^{c,n}$ and the damage driving stress ${f}_{G}^{c}$ is one-to-one mapping. In other words, the present model is only applicable for a situation that the damage evolution is monotonous while not applicable for load-unload process.

### Failure criteria

#### Failure modes

For a UD composite in planar stress state, there are five failure modes, i.e., longitudinal tension, longitudinal compression, transverse tension, transverse compression, and in-plane shear^44^. Similarly, a real stress state can be seen a combination of five stress components, i.e., longitudinal tensile and compressive normal stresses, transverse tensile and compressive normal stresses, and in-plane shear stress. A UD composite is failure when any one of the five failure modes occurs.

#### Fiber failure

The maximum normal stress criterion is often used to determine the fiber longitudinal tensile or compressive failure. A simplified criterion for fiber failure is given in Eq. (44), since longitudinal tensile or compressive failure is fiber dominant while other failure modes are matrix or interface dominant.

44 $$\{\begin{array}{ll}{\sigma }_{11}^{f} > {\sigma }_{11}^{f,ut} & when\,{\sigma }_{11}^{f}\ge 0\\ {\sigma }_{11}^{f} < -{\sigma }_{11}^{f,uc} & when\,{\sigma }_{11}^{f} < 0\end{array}$$

where ${\sigma }_{11}^{f}$ is longitudinal normal stress of a fiber, and ${\sigma }_{11}^{f,ut}$ and ${\sigma }_{11}^{f,uc}$ are longitudinal tensile and compressive strength of a fiber.

#### Matrix failure

Huang^29^ presented a generalized criterion for matrix tensile failure, which is given as Eq. (45).

45 $$\{\begin{array}{ll}{\sigma }_{I}^{m} > {\sigma }_{ut}^{m} & when\,{\sigma }_{III}^{m} < 0\\ {[{({\sigma }_{I}^{m})}^{3}+{({\sigma }_{II}^{m})}^{3}]}^{1/3} > {\sigma }_{ut}^{m} & when\,{\sigma }_{III}^{m}=0\\ {[{({\sigma }_{I}^{m})}^{3}+{({\sigma }_{II}^{m})}^{3}+{({\sigma }_{III}^{m})}^{3}]}^{1/3} > {\sigma }_{ut}^{m} & when\,{\sigma }_{III}^{m} > 0\end{array}$$

where ${\sigma }_{I}^{m},\,{\sigma }_{II}^{m},\,{\sigma }_{III}^{m}$ are three principal stresses of the matrix, and ${\sigma }_{ut}^{m}$ is matrix tensile strength.

Equation (46) is the failure criterion of matrix under compression.

46 $${\sigma }_{III}^{m} < {\sigma }_{uc}^{m}$$

where ${\sigma }_{uc}^{m}$ is matrix compressive strength.

#### Interphase failure

The interphase failure is subordinate for longitudinal tension/compression and transverse compression. However, it is critical for a UD composite under an off-axis load which is the concern of this work. As shown in Eq. (42), a Mohr-Coulomb like damage driving stress of an interphase is developed to consider the effect of transverse stress on shear damage evolution. Generally, for a UD composite under small off-axial angle load, the interphase failure is shear stress dominant. Thus, it is straightforward to take the damage driving stress as a failure criterion. Specifically, an interphase fails when its damage driving stress exceeds a critical value. However, when the off-axial angle increases, the failure mode transforms from in-plane shear to transverse failure. Thus, a maximum normal stress criterion is employed as a complementary criterion. Overall, the interphase failure criterion is given as Eq. (47).

47 $$Interphase\,fails\,when\,\{\begin{array}{ll}{\sigma }_{22}^{c} > {\sigma }_{22}^{c,ut},\, & when\,{\sigma }_{22}^{c}\ge 0\,\\ {\sigma }_{22}^{c} < {\sigma }_{22}^{c,uc},\, & when\,{\sigma }_{22}^{c} < 0\\ {f}_{G}^{c} > {f}_{G}^{c,u}\, & \end{array}$$

where ${\sigma }_{22}^{c}$ is transverse normal stress of the interphase. ${\sigma }_{22}^{c,\,ut}$ and ${\sigma }_{22}^{c,\,uc}$ are the transverse tensile and compressive strength of an interphase. ${f}_{G}^{c}$ is the damage driving stress defined in Eq. (42). ${f}_{G}^{c,u}$ is ultimate driving damage shear stress of an interphase. The three parameters ${\sigma }_{22}^{c,\,ut},\,{\sigma }_{22}^{c,\,uc},\,{f}_{G}^{c,u}$ are obtained by reverse calculation based on experimental results of off-axis loads. Equation (47) means that an interphase is failure when either the normal stress or the damage driving stress exceed its critical value.

## Experimental Validation

### Experiment data of three UD composites

To validate the present micromechanics model, experimental stress-strain curves of a series of composites are required. For the purpose of validation, experimental data of AS/PEEK^45^, IM7/8552^46,47^ and T300/7901^48^ UD carbon fiber reinforced polymer composites are employed.

**Figure 4:**
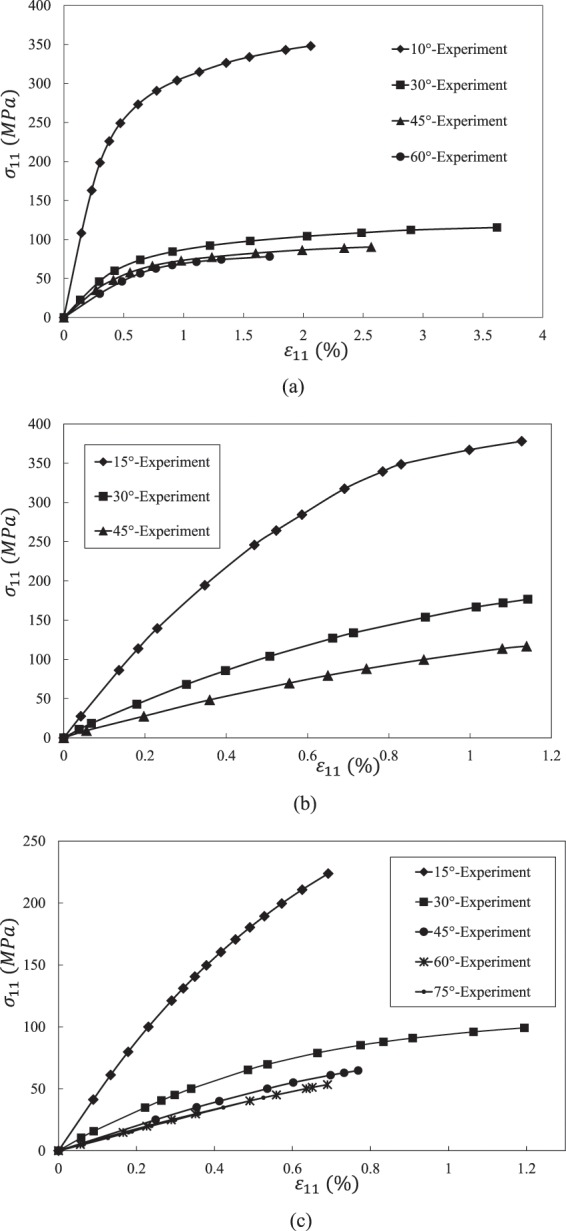
Experimental results of three UD composites under off-axial tensile loads. (**a**) AS4/PEEK UD composite. (**b**) IM7/8552 UD composite. (**c**) T300/7901 UD composite.

Table 1 lists the elastic properties of the three composites and their constituent materials. Table 2 gives the elastoplastic properties of the three epoxies. Figure 4 shows the off-axis tensile test results of the three UD composites.

**Table 1:** Elastic properties of composites and their constituents.

Composites		*E*_11_ (*GPa*)	*E*_22_ (*GPa*)	*G*_12_ (*GPa*)	*G*_23_ (*GPa*)	*υ*_12_	*Y*_*t*_ (*MPa*)
AS4/PEEK (*V*_*f*_ = 0.61)	Fiber^51^	225	15	15	7	0.2	—
	Matrix^52^	3.8	3.8	1.36	1.36	0.4	71
	Composite^45^	138	9.8	5.9	—	0.31	75
IM7/8552 (*V*_*f*_ = 0.60)	Fiber^47^	276	19	27	7	0.2	—
	Matrix^47^	4.08	4.08	1.47	1.47	0.38	99
	Composite^46^	165	8.4	5.6	2.8	0.34	73
T300/7901 (*V*_*f*_ = 0.60)	Fiber^51^	230	15	15	7	0.2	—
	Matrix^48^	3.17	3.17	1.17	1.17	0.35	85
	Composite^48^	138	8	—	—	—	40

**Table 2:** Elastoplastic properties of the three epoxies.

Epoxy	Elastoplastic properties									
PEEK^45^	${\sigma }_{Y}^{m,n}$ (MPa)	25	36	45	52	58	63	65	67	71
	${E}_{T}^{m,n}$ (GPa)	3.8	3.06	2.74	2.06	1.64	0.8	0.52	0.37	0.2
8552^a^^47^	${\tau }_{Y}^{m,n}$ (MPa)	12	21	29	39	48	52	56	57	
	${G}_{T}^{m,n}$ (GPa)	1.45	1.34	1.25	1.13	0.96	0.81	0.71	0.63	
7901^48^	${\tau }_{Y}^{m.n}$ (MPa)	20	33	39	43	45	47	49	51	55
	${G}_{T}^{m,n}$ (GPa)	0.8	0.67	0.61	0.53	0.47	0.43	0.39	0.33	0.12

^a^Use property of 8551-7 epoxy due to lack of experimental data of 8552 epoxy.

### Interphase properties

Before the interphase properties is involved, the value of the bridging parameters *α*, *β* must be determined. In general, the *α*, *β* are set as 0.3 for most polymeric fibrous composites. Nevertheless, it is better to calibrate the *α*, *β* according to experimental data when available. In this work, they can be adjusted by fitting the predicted elastic properties of a composite to measured ones.

In addition, the volume fraction of an interphase should also be set beforehand. As reported by many researchers^35,36^, the interface damage can be simulated by an interphase stiffness degradation. In other words, the interphase is an approximation of an interface but not means a real interphase material. The interface thickness is zero while the interphase thickness is non-zero. If a linear-spring interface model is employed, there exists relations among the interface properties, interphase properties, and the interphase thickness, as shown in the equation below.

48 $${k}_{n}=\frac{{t}^{c}}{{\lambda }^{c}+2{G}^{c}},\,{k}_{t}=\frac{{t}^{c}}{{G}^{c}}$$

*k*_*n*_ and *k*_*t*_ are normal and tangent elastic constants of an interface model, and *t*^*c*^, *λ*^*c*^, and *G*^*c*^ are thickness and Lame’s constants of an interphase model. From the equation, it can be found that, the interphase properties vary with the interphase thickness for a target interface model. However, the values of the interphase properties won’t affect the interface model, as long as the relation between the interphase thickness and interphase properties remain unchanged. Thus, for a composite, one can define the interphase thickness arbitrarily in a reasonable range, and then calculate the interphase properties from the interface parameters. According to Mikata *et al*.^49^ and Benveniste *et al*.^50^, a general interphase volume fraction ranges from 1% to 10%. Further, it is easier to reversely calculate interphase properties with a larger interphase volume fraction. Thus, for the three composites involved in this work, the authors reasonably set all the interphase volume fractions as 10%, although their interface parameters may be different.

Since there are two independent variables in the interphase mode, the shear modulus ${G}_{D}^{c}$ and the coupling parameter *b*, two off-axis stress-strain curves are necessary to determine the ${f}_{G}^{c}-{G}_{D}^{c}$ relation of an interphase. In order to obtain ${G}_{D}^{c,n}$, a shear dominant small off-axis angle test is needed. To obtain *b*, a 45° off-axis stress-strain curve is advisable.

Following the reverse calculation process shown in Fig. 3, parameters involved in the interphase model can be obtained. Table 3 lists the pre-determined parameters and experimental curves required in the reverse calculation and model validation process.

**Table 3:** Parameters of reverse calculation process.

Composites	Bridging parameters	Interphase volume fraction *V*^*c*^	Curves for reverse calculation	Curves for model validation
AS4/PEEK	α = β = 0.3	10%	10°, 45°	30°, 60°
IM7/8552	α = β = 0.3	10%	15°, 45°	30°
T300/7901	α = 0.4, β = 0.5	10%	15°, 45°	30°, 60°, 75°

**Figure 5:**
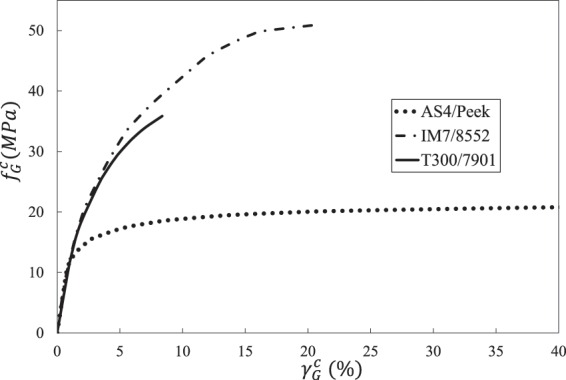
Reversely calculated interphase properties of three UD composites.

Results of the reverse calculation process are shown in Fig. 5. It should be noted that the shear strain in Fig. 5 is an equivalent shear strain but not the regular one defined in elasticity. Its expression is given as Eq. (49).

49 $$d{\gamma }_{G}^{c}=d{f}_{G}^{c}/{G}_{D}^{c,n}$$

where $d{f}_{G}^{c}$ is an incremental shear damage driving stress, ${G}_{D}^{c,n}$ is an instantaneous shear moduli in the *n*^*th*^ load step. For the convenience of engineering application, an empirical analytical model for an interphase damage property is desirable. It is found that the three curves are exponential function like. Thus, an empirical model is developed in Eq. (50), where linear functions are employed for the undamaged part and the part around failure. An exponential function is used for the damaged part.

50 $${f}_{G}^{c}=\{\begin{array}{ll}{G}^{c}{\gamma }_{G}^{c}, & {\gamma }_{G}^{c}\le {\gamma }_{G}^{c,e}\\ {\alpha }_{G}{e}^{{\beta }_{G}{\gamma }_{G}^{c}}+{\lambda }_{G}, & {\gamma }_{G}^{c,e} < {\gamma }_{G}^{c}\le {\gamma }_{G}^{c,p}\\ {G}_{u}^{c}({\gamma }_{G}^{c}-{\gamma }_{G}^{c,p})+{\xi }_{G}, & {\gamma }_{G}^{c} > {\gamma }_{G}^{c,p}\end{array}$$

In Eq. (50), *G*^*c*^ is an elastic shear modulus of an interphase. ${G}_{u}^{c}$ is the tangential shear modulus of an interphase when it approaches failure. ${\gamma }_{G}^{c,e}$ is an elastic ultimate equivalent shear strain of an interphase. ${\gamma }_{G}^{c,p}$ is the equivalent shear strain of an interphase at the intersection point of the exponential curve and the following linear curve. ${\alpha }_{G},{\beta }_{G},{\lambda }_{G}$, and *ξ*_*G*_ are undetermined coefficients. An exponential function form is selected from two perspectives. On the one hand, the function is empirical and phenomenological. In other words, the exponential function is used because the reverse calculated results are indeed exponential like. Certainly, researchers can develop other functions for the interphase degradation based on their own observation and calculation. The function form will not affect the essence of the elastoplastic damage model developed in this work. On the other hand, theoretically, the interphase shear modulus should gradually degrade with the damage evolution, and it approaches to a certain value eventually. Thus, an exponential function is better to fit this kind of trend, compared to other functions, such as a polynomial or logarithmic function.

Please note that, all the parameters involved in Eq. (50) can be determined by the same two curves used for calibrating ${f}_{G}^{c}-{G}_{D}^{c}$ relation. Thus, no more extra experimental data is needed. Table 4 lists the empirical interphase models of the three composites obtained by the reverse calculation process.

**Table 4:** Empirical interphase models.

Composites		Empirical equations (MPa)
AS4/PEEK	*b* = 0.2	$${f}_{G}^{c}=\{\begin{array}{ll}1357\,{\gamma }_{G}^{c}, & {\gamma }_{G}^{c}\le 0.84 \% \\ -11\,{e}^{-26.8{\gamma }_{G}^{c}}+20.2, & 0.84 \% < {\gamma }_{G}^{c}\le 21 \% \\ 3({\gamma }_{G}^{c}-0.21)+20.2 & {\gamma }_{G}^{c} > 21 \% \end{array}$$
	${f}_{G}^{c,u}=21.8\,MPa$ ${\sigma }_{22}^{c,ut}=43\,\text{MPa}$	
IM7/8552	*b* = 0.8	$${f}_{G}^{c}=\{\begin{array}{ll}1478\,{\gamma }_{G}^{c}, & {\gamma }_{G}^{c}\le 0.55 \% \\ -48.3\,{e}^{-17.5{\gamma }_{G}^{c}}+52, & 0.55 \% < \,{\gamma }_{G}^{c}\le 16 \% \\ 24({\gamma }_{G}^{c}-0.16)+49 & \,{\gamma }_{G}^{c} > 16 \% \end{array}$$
	${f}_{G}^{c,u}=51$ ${\sigma }_{22}^{c,ut}=35\,MPa$	
T300/7901	*b* = 0.85	$${f}_{G}^{c}=\{\begin{array}{ll}1174\,{\gamma }_{G}^{c}, & {\gamma }_{G}^{c}\le 1 \% \\ -35.8\,{e}^{-27.4{\gamma }_{G}^{c}}+39, & 1 \% < {\gamma }_{G}^{c}\le 8 \% \\ 140({\gamma }_{G}^{c}-0.08)+35 & {\gamma }_{G}^{c} > 8 \% \end{array}$$
	${f}_{G}^{c,u}=39$ ${\sigma }_{22}^{c,ut}=33\,MPa$	

### Experimental validation and discussion

**Figure 6:**
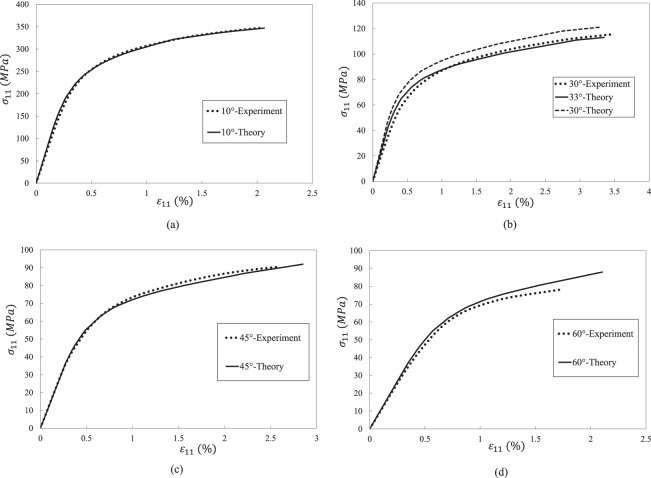
Stress-strain curves of an AS4 PEEK UD composite under off-axial tension. (**a**) 10° off-axial tension. (**b**) 30° off-axial tension. (**c**) 45° off-axial tension. (**d**) 60° off-axial tension.

**Figure 7:**
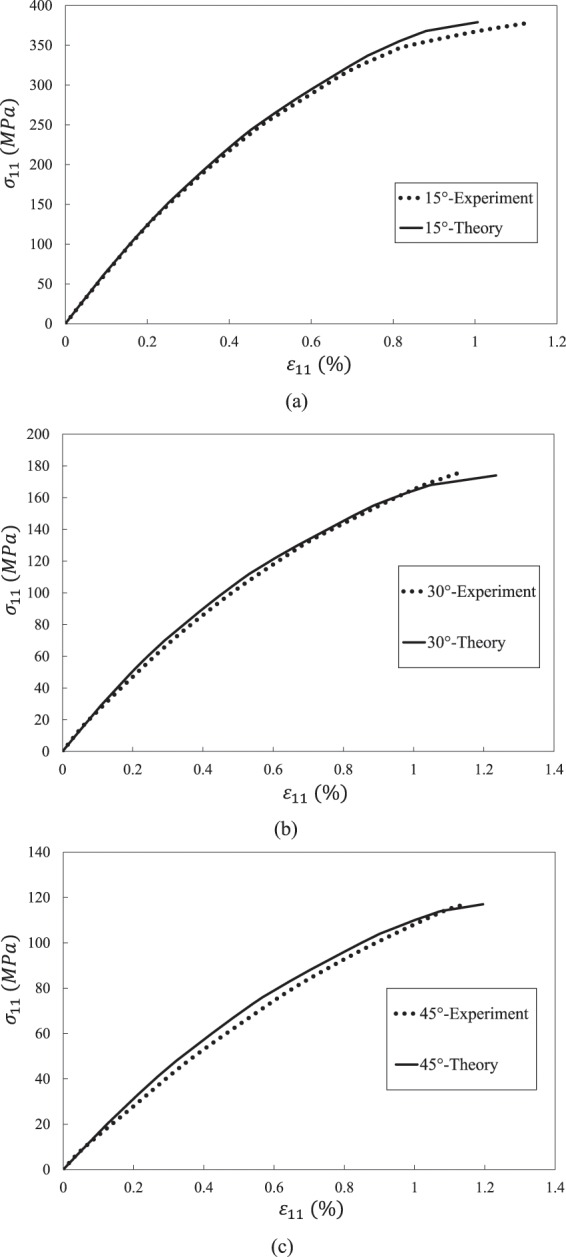
Stress-strain curves of an IM78552 UD composite under off-axial tension. (**a**) 15° off-axial tension. (**b**) 30° off-axial tension. (**c**) 45° off-axial tension.

**Figure 8:**
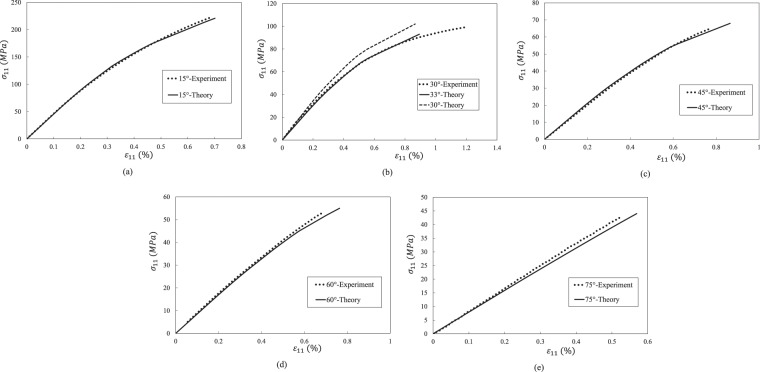
Stress-strain curves of an T300 7901 UD composite under off-axial tension. (**a**). 15° off-axial tension. (**b**) 30° off-axial tension. (**c**) 45° off-axial tension. (**d**) 60° off-axial tension. (**e**) 75° off-axial tension.

As shown in Table 3, two off-axis curves of a composite are used for reverse calculation of interphase properties. Other off-axial curves are used as validation. Figures 6–8 illustrate the comparison between theoretical and experimental stress-strain curves. Tables 5–7 are strength prediction results of three composites.

**Table 5:** Strength prediction results of AS4/PEEK UD composite.

	10°		30°		45°		60°	
	Strength	Strain	Strength	Strain	Strength	Strain	Strength	Strain
Experiment	348 MPa	2.06%	115.5 MPa	3.46%	90.4 MPa	2.57%	78 MPa	1.72%
Theory	347 MPa	2.064%	121 MPa (30°) 113 MPa (33°)	3.27% (30°) 3.34% (33°)	92 MPa	2.85%	88 MPa	2.1%
Error	0.3%	0.2%	4.8% (30°) 1.3% (33°)	5.5% (30°) 6% (33°)	1.8%	11%	13%	22%

**Table 6:** Strength prediction results of IM7/8552 UD composite.

	15°		30°		45°	
	Strength	Strain	Strength	Strain	Strength	Strain
Experiment	378 MPa	1.13%	176.6 MPa	1.14%	116.8 MPa	1.14%
Theory	379 MPa	1%	174 MPa	1.23%	117 MPa	1.19%
Error	0.3%	2.7%	1.5%	7.9%	0.2%	4.4%

**Table 7:** Strength prediction results of T300/7901 UD composite.

	15°		30°		45°		60°		75°	
	Strength	Strain	Strength	Strain	Strength	Strain	Strength	Strain	Strength	Strain
Experiment	224 MPa	0.69%	99 MPa	1.19%	65 MPa	0.77%	53 MPa	0.69%	43 MPa	0.53%
Theory	223 MPa	0.71%	102 MPa (30°) 93 MPa (33°)	0.87% (30°) 0.89% (33°)	68 MPa	0.86%	58 MPa	0.82	44 MPa	0.57%
Error	0.4%	2.9%	3% (30°) 6.3% (33°)	27% (30°) 25% (33°)	4.9%	11.7%	8.6%	19%	2.8%	7.5%

Overall speaking, it is found from Figs. 6–8 that the predicted results agree well with experimental data. The predicted nonlinear behavior of all the cases shown in Figs. 6–8 are coincident to experimental data, which proves the model’s capability on evaluating nonlinear deformation of UD composite under off-axial tension. When focusing on the off-axial tensile strength, most of the strength prediction errors are less than 10%. Only the strength error of AS4/PEEK UD composite under 60° is slightly larger than 10%, which is also good enough. Thus, it is evident that the model performs good in strength prediction. When it comes to the ultimate strain, the prediction errors become larger, but most cases are still satisfied (less than 15%).

For the case of 30° off-axial tension, the prediction result of IM7/8552 UD composite is in high accuracy compared with experimental data. Only the tangential modulus slightly deviates from the experimental value around the failure point, which is not critical for engineering application. However, there are some deviations between the predicted and experimental curves of AS4/PEEK and T300/7901 UD composites under 30° off-axial tension. Such deviations may result from the angle error induced by manual lay-up process. Thus, an off-axial angle of 33° instead of 30° is used for the prediction of the two cases. Then, good correlations are achieved for the nonlinear deformation of the two composites. Nevertheless, the ultimate error in Fig. 8(b) is still not ignorable. It may because the stress based failure criteria is employed. When a material approaches failure, a slight variation of stress may introduce to a significant strain fluctuation. Overall, it should be noted that the ultimate strength and strain are acceptable whether 30° or 33° is used, considering potential source of experimental error.

For the cases of Fig. 6(d), the strength prediction accuracy is acceptable. However, the error of the ultimate strain is relative large. The error may result from three aspects. Firstly, the stress-based failure criterion may be not able to capture the strain variation when a material is approach failure. Secondly, the developed interphase model assumes that the transverse Young’s modulus of an interphase, *E*^*c*^, keeps elastic throughout a load case. However, in fact, *E*^*c*^ may also degrade with the damage evolution of an interphase. The degradation of *E*^*c*^ of AS4/PEEK may be more significant than the other two composites, since the PEEK epoxy is more ductile than the other two epoxies. Lastly, when the off-axial angle becomes large, the failure mode gradually transferred from in-plane failure dominant to tension failure. For T300/7901 UD composite, the coupling parameter *b* is 0.85, indicating that the effect of transverse stress on failure can be fully considered. However, the engineering reversed coupling parameter *b* for AS4/PEEK is 0.2, meaning that the effect of transverse stress may be underestimated. Nevertheless, the prediction error is still reasonable (13% for strength and 22 for ultimate strain), considering the fact that the experimental scatter of a composite is generally much larger than a homogeneous material.

The model developed in this work incorporates the contribution of interface damage evolution and matrix elastoplasticity on the nonlinear behavior of a composite. A Mohr-Coulomb like failure criterion is proposed for the stiffness degradation of an interphase, with which the effect of transverse normal stress on the shear property can be considered. Theoretically, the current model is applicable for a UD composite under off-axial tension and compression. However, due to the lack of experimental data, the model is only assessed for UD composites under off-axial tension. For the nonlinear behavior and ultimate strength, the current model provides satisfied prediction results. For the ultimate strain, the prediction error becomes larger but still in a reasonable range. Thus, overall speaking, it is believed that the model is applicable for UD composites under off-axial tension. It also has potential applicability on off-axial compression cases, once validated by experiments. In addition, further investigation on the degradation of Young’s modulus and other failure criteria will be made in future works.

## Conclusions

An analytical micromechanics model with matrix elastoplasticity and interface damage is developed for the simulation of nonlinear behavior of a composite. In the model, an interphase with stiffness degradation is employed to simulate the progress interface damage in a composite. For the convenience of engineering application, an empirical model is present to describe the relationship between the shear damage driving stress and the damage property of an interphase. The model is validated by a series of off axial tension tests of three kinds of UD composites. In addition, owing to the explicit and analytical form, the model is of high computational efficiency and convenient to be applied in engineering.
